# Prediction of pulmonary metastasis in esophageal carcinoma patients with indeterminate pulmonary nodules

**DOI:** 10.1186/s12957-023-03211-6

**Published:** 2023-10-09

**Authors:** Maohui Chen, Hongjin Wang, Yizhou Huang, Feilong Guo, Wei Zheng, Chun Chen, Bin Zheng

**Affiliations:** 1 Department of Thoracic Surgery, Fujian Medical University Union Hospital, Fuzhou, China; 2 Key Laboratory of Cardio-Thoracic Surgery (Fujian Medical University), Fujian Province University, Fujian, China; 3 National Key Clinical Specialty of Thoracic Surgery, Fuzhou, China; 4 Department of Cardiovascular Surgery, Longyan First Affiliated Hospital of Fujian Medical University, Longyan, China

**Keywords:** Esophageal squamous cell carcinoma, Indeterminate pulmonary nodules, Pulmonary metastasis, Prognostic factor, Predictive model

## Abstract

**Background:**

Indeterminate pulmonary nodules (IPNs) are common after surgery for esophageal cancer. The paucity of data on postoperative IPNs for esophageal cancer causes a clinical dilemma.

**Objective:**

The aim of this study was to identify the characteristics and clinical significance of IPNs after radical esophagectomy for metastatic esophageal cancer, determine the risk factors for pulmonary metastasis, and construct a risk score model to standardize the appropriate time to either follow up or treat the patient.

**Methods:**

All consecutive patients with esophageal squamous cell carcinoma (ESCC) who underwent radical surgery between 2013 and 2016 were included in this retrospective study. Univariate and multivariate logistic regression analyses were performed to identify independent risk factors and develop risk score models.

**Results:**

A total of 816 patients were enrolled in the study. During a median follow-up period of 45 months, IPNs were detected in 221 (27.1%) patients, of whom 66 (29.9%) were diagnosed with pulmonary metastases. The following five variables maintained prognostic significance after multivariate analyses: the pathologic N category, number of IPNs, shape of IPNs, time of detection of IPNs, and size of IPNs. The Pulmonary Metastasis Prediction Model (PMPM) scale ranges from 0 to 15 points, and patients with higher scores have a higher probability of pulmonary metastases. The Hosmer–Lemeshow test showed a good calibration performance of the clinical prediction model (*χ*^2^ = 8.573, *P* = 0.380). After validation, the PMPM scale showed good discrimination with an AUC of 0.939.

**Conclusion:**

A PMPM scale for IPNs in patients who underwent esophagectomy for ESCC may be clinically useful for diagnostic and therapeutic decision-making.

## Background

Esophageal cancer is the seventh most prevalent malignancy in humans and the sixth leading cause of tumor-related death worldwide [1]. Surgical resection remains the primary treatment as it offers effective, sustained remission and the best chance of cure. However, the 5-year survival rate in most countries rarely exceeds 40% as the postoperative recurrence rate is high [2, 3]. The lungs are one of the common sites of metastatic deposition of malignant tumors, which occurs at a rate of between 8.6 and 12.1% because they are highly vascularized and have ample lymphatic drainage [4–6].

Owing to benign lesions such as inflammation, tuberculosis, and mycobacteria also present as pulmonary nodules and are more common, resulting in lung metastases being different from metastases to other organs. Early lung metastases may present as single or multiple pulmonary nodules that are not easily distinguished from the lesions described previously, which causes a management dilemma [7]. Mai Hanamiya et al. showed that out of 308 patients with extra-pulmonary malignancies, IPNs were detected in 233 (75.6%) patients, and 137 of them were followed up and only 28 showed malignancy [8].

For postoperative pulmonary metastatic lesions in esophageal cancer, early diagnosis and treatment, including surgical resection, are essential to improve the patient’s prognosis [9–11]. However, current guidelines for esophageal cancer, such as the National Comprehensive Cancer Network (NCCN) guidelines, American Joint Committee on Cancer (AJCC) guidelines, and the Japanese Classification of Esophageal Cancer, do not address the diagnostic approach and risk factors for pulmonary metastases of esophageal cancer, and there are no relevant large-scale studies with high levels of evidence [12–15]. The current diagnostic procedures for lung metastases are still invasive techniques such as percutaneous lung puncture or surgical resection; however, these techniques are not recommended for patients in the early stages of lung metastasis, as these patients may present with only a single or multiple nodules on CT, which is difficult to pinpoint at this time, making puncture biopsy difficult and low in accuracy.

Therefore, we designed this study to collect the clinical data of patients with new postoperative pulmonary nodules, investigate the risk factors for pulmonary metastasis, and construct a risk score model to standardize the appropriate time to follow up or perform treatments.

## Methods

### Patients

We retrospectively collected clinical data, radiologic data, and follow-up results of patients with esophageal squamous cell carcinoma who underwent surgery at the Department of Thoracic Medicine, Union Hospital of Fujian Medical University, from January 2013 to December 2016. The inclusion criteria for this study were as follows: (1) patients who underwent radical (R0) resection with systemic lymph node dissection, (2) postoperative pathological diagnosis of esophageal squamous cell carcinoma, (3) regular follow-up and ability to provide regular radiologic data, and (4) IPNs were detected during postoperative follow-up. Exclusion criteria for this study were as follows: (1) diagnosis of another type of malignancy, (2) confirmed extra-pulmonary metastasis, and (3) presence of a highly suspicious recurrent metastatic lesion outside the lung, with or without receiving appropriate treatment. We defined pathological staging according to the International Union for Cancer Control 8th edition staging system for esophageal cancer [13].

### Follow-up evaluation

For asymptomatic patients, each review included a complete history-taking, laboratory testing, computed tomography (CT) of the neck, chest, and upper abdomen, and abdominal and cervical lymph node ultrasound. For patients with IPNs on CT, blood sampling, sputum bacterial cultures, sputum fungal cultures, as well as other tests related to fungal infections and tuberculosis were recommended. In cases of diagnostic difficulties, respiratory medicine consultation or multidisciplinary consultation was also necessary. If metastases were considered, systemic investigations such as CT, magnetic resonance imaging (MRI), and positron emission tomography–computed tomography (PET-CT), and percutaneous pulmonary puncture biopsy, and, if necessary, thoracoscopic surgical resection, were recommended. If non-metastatic lesions were considered, a repeat CT of the lungs is routinely recommended in 2 or 3 months. The time of detection of IPNs was defined as the interval between surgery and the appearance of IPNs. All postoperative CT and other images were reviewed individually by the two senior experts. The diagnosis of pulmonary metastases included pathology and radiography, which require the progressive multiplication and enlargement of IPNs after at least two CT reviews and at least 6 months of follow-up [8, 16].

### Clinical features

Data on the following clinical characteristics were collected: age, sex, history of previous pulmonary infections (tuberculosis, fungal, etc.), tumor location of ESCC, surgery approach, the range of lymph node dissection, pathologic T category, pathologic N category, and tumor differentiation.

### Radiologic characteristic data

Radiologic characteristics included the time of detection of IPNs, the number of IPNs, the location of the largest IPN (left lobe or right lobe; upper, middle, or lower), the area of the largest IPN (outer 1/3 of the lung, middle 1/3 of the lung, and inner 1/3 of the lung, depending on the distance between the nodule and the lung’s surface), the size of the largest IPN, the shape of the largest IPN, the calcification of the largest IPN, and the mediastinal lymph node enlargement.

### Statistical analysis

Baseline patient characteristics were compared using the analysis of variance for continuous variables and the chi-square test for discrete variables. Univariate and multivariate logistic regression analyses were used to identify independent predictors of the probability of pulmonary metastases. Statistically significant variables (*P* < 0.05) from the multivariate logistic regression analysis were entered into the predicted model. Based on the results of the multivariate logistic regression analyses. Scores for each variable of the PMPM scale were calculated based on the regression coefficient values (*β* values) taken as natural numbers. The clinical prediction model for pulmonary metastasis of IPNs expresses the probability of metastasis as a function of the five variables, as follows: (1) probability of pulmonary metastasis = eX/(1 + ex), where *e* is the base of natural logarithms; (2) *x* =  − 9.623 + (model score of pathologic N category) + (model score of number of IPNs) + (model score of shape of largest IPN) + (model score of time of detection of IPNs) + (model score of size of the largest IPN). The models were evaluated by the areas under the ROC curve. The scores for the different variables were summed up to yield a total score for each patient, which could be converted to a predicted probability of pulmonary metastasis. All patients were scored according to this PMPM scale, with a histogram showing the probability of pulmonary metastasis for each score, and risk-stratified by the magnitude of the probability of metastasis.

## Results

### Characteristics of indeterminate pulmonary nodules

During a median follow-up period of 45 (range, 1–107) months, IPNs were detected in 221 (27.1%), of which 66 (29.9%) were diagnosed with pulmonary metastases. Forty patients had metastases confirmed by histopathology, and 26 were diagnosed via radiography during follow-up. The clinical and radiologic characteristics of the study subjects are presented in Table 1. The mean age was 58.17 ± 8.18 years, and males (75.1%) were predominant. Only 9 (4.1%) patients had a previous history of specific lung infections. The most common tumor location was the middle thoracic segment in 133 (60.2%), followed by the lower thoracic segment in 67 (30.3%), and finally, the upper thoracic segment in 21 (9.5%). The majority (96.8%) of patients underwent minimally invasive radical esophageal cancer, and 173 (78.3%) patients underwent two-field lymph node dissection. A total of 29 (13.1%) patients received neoadjuvant preoperative treatment, of whom 19 (8.6%) received preoperative neoadjuvant chemotherapy and 10 (4.5%) received neoadjuvant chemoradiotherapy. Postoperative adjuvant chemotherapy was given to 104 (47.1%) patients, and postoperative adjuvant chemoradiotherapy was given to 19 (8.6%) patients. The most common pathological *T* stage was T3 with 104 cases (47.1%), and N0 was the most common pathological *N* stage with 119 cases (53.8%). The mean time of detection of IPN was 22.86 ± 16.89 months and 11.44 ± 13.13 months in the pulmonary metastasis group and non-pulmonary metastasis group, respectively. At least three nodules (multiple IPNs) were found in 90 of 221 patients. Metastatic lesions presented predominately (60 cases, 90.1%) as solid nodules, while in non-metastatic lesions, ground-glass or partially ground-glass was prevalent (97 cases, 62.3%). The most common site of the largest IPN was the right upper lobe (77 cases, 34.8%), followed by the right lower lobe (47 cases, 21.3%), left upper lobe (42 cases, 19.0%), and left lower lobe (36 cases, 16.3%), and the least common was the right middle lung (19 cases, 8.6%). The majority of nodules in the metastatic group (63.6%) were found in the outer 1/3 of the lung field. Calcification occurred in 27 (12.2%) non-metastatic nodules, and no malignant nodules were present. Almost all (97.0%) metastatic nodules were round or round-like in shape while 39.4% of non-metastatic nodules had irregular shapes. On lung CT examination, enlarged mediastinal lymph nodes were found in a total of 32 patients, 25 in the metastatic group and 7 in the non-metastatic group.

**Table 1:** Clinical characteristics and radiologic characteristics of the 221 patients with IPNs after esophagectomy

Variable	Total *n* = 221	PM *n* = 66	Non-PM *N* = 155	*p* value
Age(years)	58.17 ± 8.18	58.76 ± 8.22	57.92 ± 8.18	0.992
Sex				0.628
Male	166	51	115	
Female	55	15	40	
History of previous lung infections				0.546
Yes	9	4	5	
No	212	62	150	
Tumor location of ESCC				0.018
Upper 1/3	21	5	16	
Middle 1/3	133	49	84	
Lower 1/3	67	12	55	
Pathologic T category				0.413
1	55	14	41	
2	50	12	38	
3	104	35	69	
4	12	5	7	
Pathologic N category				< 0.001
0	119	16	103	
1	52	20	32	
2	18	12	10	
3	32	18	10	
Tumor differentiation				0.051
Well(G1)	80	17	63	
Moderate(G2)	114	37	77	
Poor(G3)	27	12	15	
The range of lymph node dissection				0.405
Mediastinal + abdominal	173	54	119	
Mediastinal + abdominal cervical	48	12	36	
Surgery approach				0.731
VATS	214	63	151	
Open surgery	7	3	4	
Neoadjuvant therapy				0.201
None	192	57	135	
Chemotherapy	19	8	11	
Chemoradiotherapy	10	1	9	
Postoperative therapy				< 0.001
None	98	14	84	
Chemotherapy	104	41	63	
Chemoradiotherapy	19	11	8	
Location of the largest IPN				0.923
Right upper lobe of the lung	77	22	55	
Right middle lobe of the lung	19	6	13	
Right lower lobe of the lung	47	16	31	
Left upper lobe of the lung	36	9	27	
Left lower lobe of the lung	42	13	29	
Time of detection of IPNs ( year)	14.85 ± 15.25	22.86 ± 16.89	11.44 ± 13.13	< 0.001
≤ 1	131	22	109	
> 1, ≤ 2	42	16	26	
> 2	48	28	20	
Number of IPNs				< 0.001
< 3	131	5	126	
≥ 3	90	61	29	
Size of the largest IPN (mm)				< 0.001
≤ 5	72	1	71	
> 5, ≤ 10	86	21	65	
> 10, ≤ 30	58	40	18	
> 30	5	4	1	
Density of the largest IPN				< 0.001
Solid nodules	118	60	58	
Ground glass nodules or mixed ground glass nodules	103	6	97	
Shape of the largest IPN				< 0.001
Round or round-like	158	64	94	
Others	63	2	61	
Area of the largest IPN				0.342
Inner 1/3	29	10	19	
Middle 1/3	90	22	68	
Outer 1/3	102	34	68	
Calcification of the largest IPN				< 0.001
Yes	27	0	27	
No	194	66	128	
Mediastinal lymph nodes enlargement				< 0.001
No	189	41	148	
Yes	32	25	7	

*IPN* indeterminate pulmonary nodule, *ESCC* esophageal squamous cell carcinoma, *VATS* video-assisted thoracoscopic surgery

### Univariate and multivariate analyses

Univariate and multivariate analyses of potential predictors of IPNs are summarized in Table 2. The pathologic N category, size of the largest IPN, time of detection of IPNs, number of IPNs, shape of the largest IPN, and mediastinal lymph node enlargement and density of the largest IPN were associated with the malignant nature of SPL. However, only the pathologic N category, size of the largest IPN, time of detection of IPNs, number of IPNs, and shape of the largest IPN remained as independent indicators of pulmonary metastasis after the multivariate regression analysis (Table 3). The Hosmer–Lemeshow test showed a good calibration performance (*χ*^2^ = 8.573, *P* = 0.380).

**Table 2:** Univariable analysis and multivariate regression analysis of the 221 patients with IPNs after esophagectomy

Multivariable analysis			
Variable	Univariable Analysis *P*	Hazard ratio	*p*
Pathologic N category	< 0.001		0.010
0		Reference	
1		2.542	
2		6.805	
3		25.118	
Number of IPNs	< 0.001		< 0.001
< 3		Reference	
≥ 3		27.180	
Shape of the largest IPN	< 0.001		0.008
Round or round-like		8.949	
Others		Reference	
Time of detection of IPNs(year)	< 0.001		0.041
< 1		Reference	
1–2		4.088	
> 2		4.388	
Size of the largest IPN	< 0.001		0.003
< 5 mm		Reference	
5–10 mm		22.397	
10–30 mm		59.608	
> 30 mm		302.156	
Mediastinal lymph node enlargement	< 0.001	0.644	0.586
Density of the largest IPN	< 0.001	1.975	0.336
Age	0.487		
Gender	0.628		
History of lung infection	0.337		
Surgery approach	0.451		
The range of lymph node dissection	0.406		
Pathologic T category	0.422		
Tumor differentiation	0.056		
Location of the largest IPN	0.924		
Area of the largest IPN	0.345		
Calcification of the largest IPN	0.998		

*IPN* indeterminate pulmonary nodule

**Table 3:** Multivariate regression analysis for the Pulmonary Metastasis Prediction Model

Multivariable analysis		
Variable	*β* value	*p*
Pathologic N category		0.010
0	Reference	
1	0.992	
2	1.831	
3	3.196	
Number of IPNs		< 0.001
< 3	Reference	
≥ 3	3.317	
Shape of the largest IPN		0.008
Round or round-like	2.355	
Others	Reference	
Time of detection of IPNs(year)		0.041
< 1	Reference	
1–2	1.476	
> 2	1.659	
Size of the largest IPN		0.003
< 5 mm	Reference	
5–10 mm	3.274	
10–30 mm	4.482	
> 30 mm	6.341	

*IPN* indeterminate pulmonary nodule

### Pulmonary Metastasis Prediction Model score

**Fig. 1:**
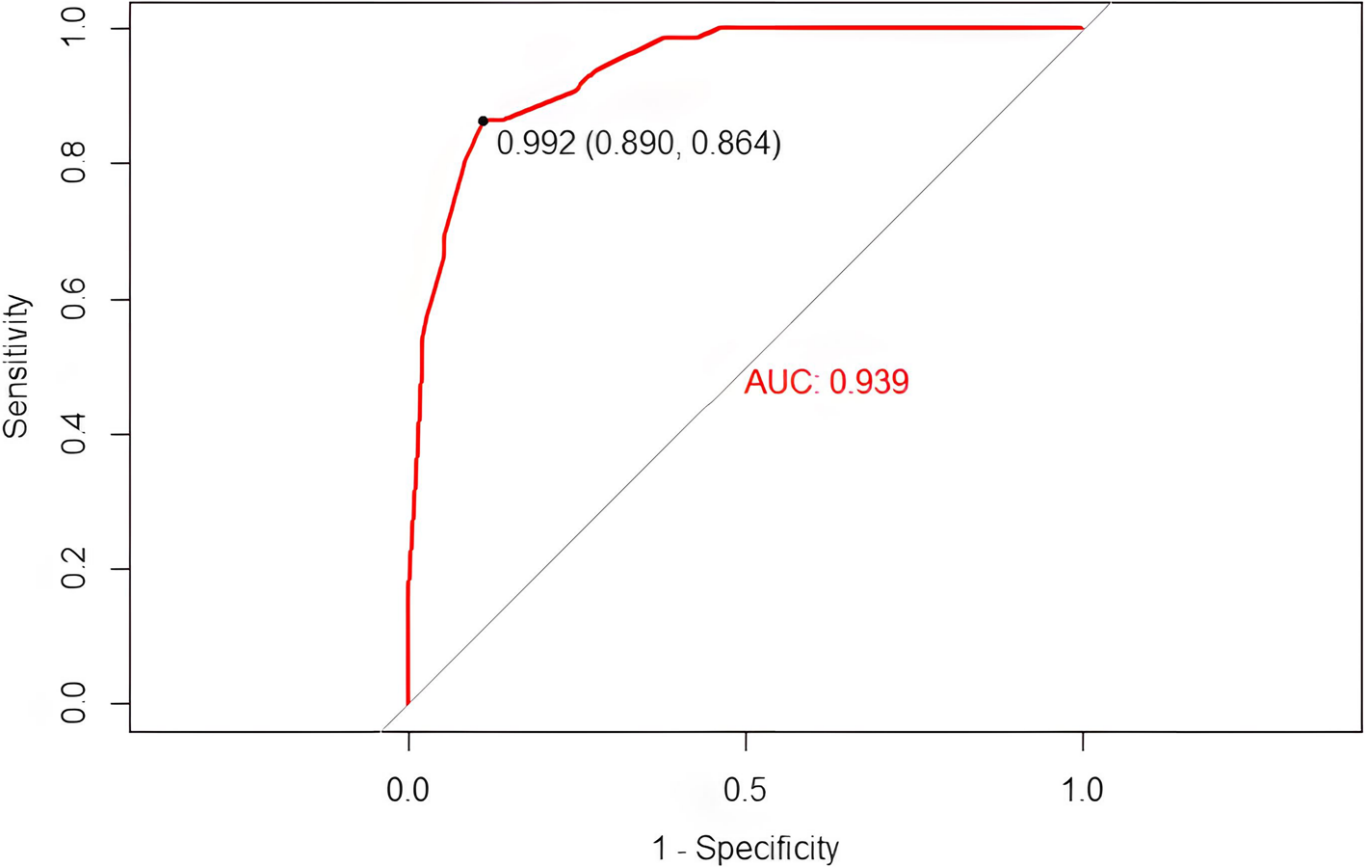
ROC curve of the Pulmonary Metastasis Prediction Model scale

**Fig. 2:**
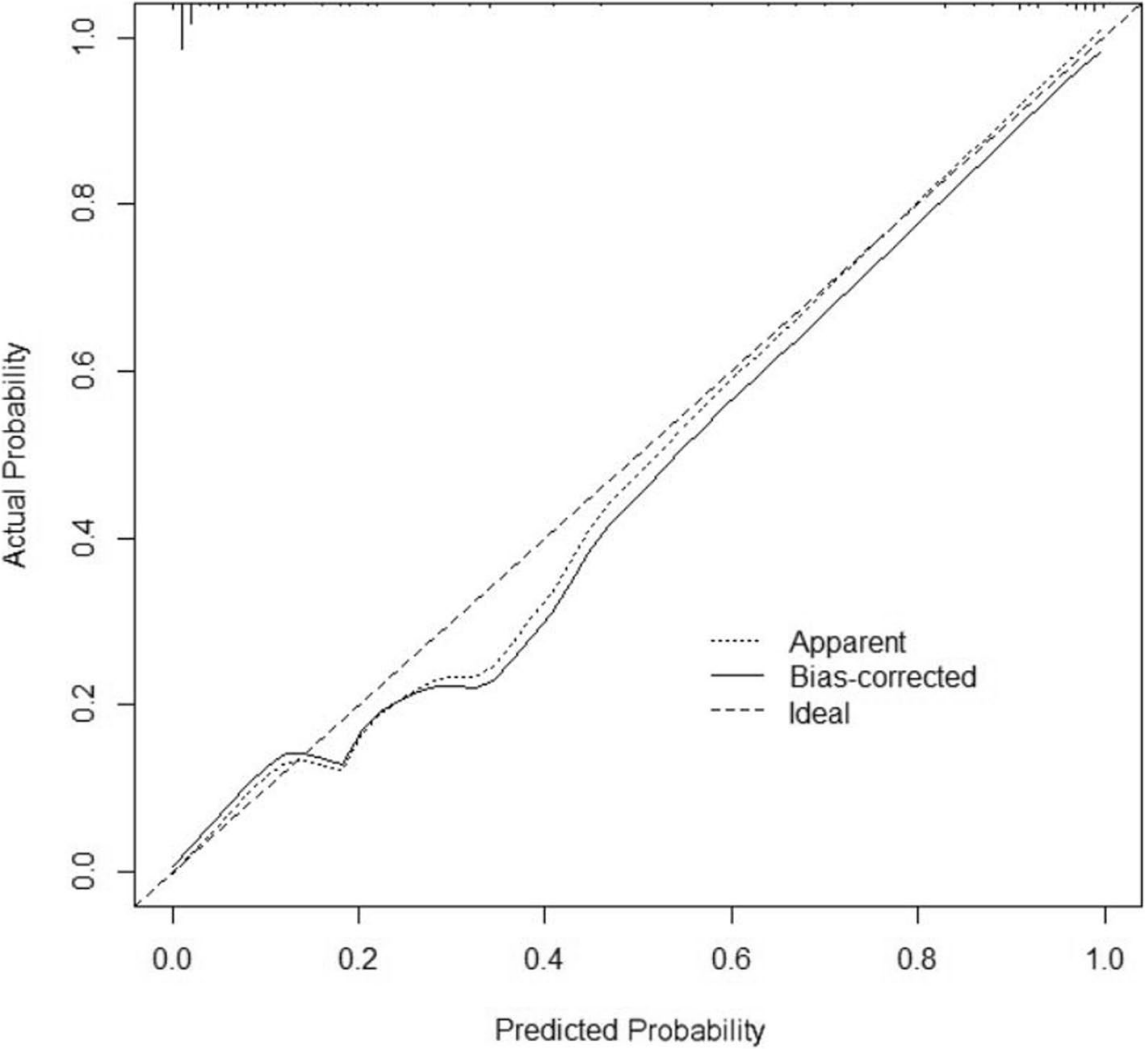
The calibration curve of the Pulmonary Metastasis Prediction Model scale

**Fig. 3:**
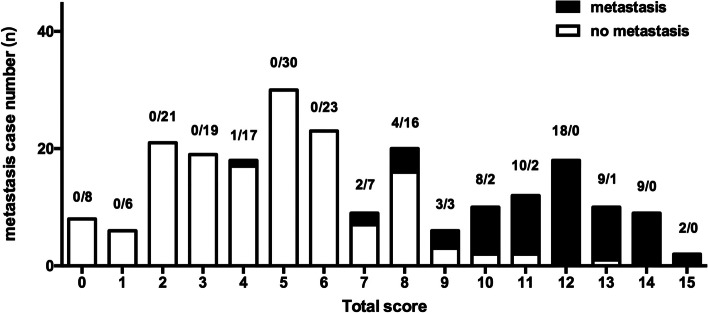
Distribution of 221 new patients with IPNs based on the Pulmonary Metastasis Prediction Model scale

A Pulmonary Metastasis Prediction Model score was established according to *β* regression coefficients estimated from the logistic regression model (Tables 3 and 4). The score ranges from 0 to 15 points, and patients with higher scores have a higher probability of pulmonary metastasis. The C-index of the diagnostic model was 0.973. According to ROC analysis, the score showed good discrimination with an AUC of 0.946 (Fig. 1). The observed results were consistent with the predicted outcomes based on the calibration curve (Fig. 2). A histogram (Fig. 3) showing a visual inspection of the probability of pulmonary metastasis for each score and the probability of pulmonary metastasis based on the Pulmonary Metastasis Prediction scale is shown in Table 5. The risk of pulmonary metastasis is very low (0.8%) at scores 0–6, while it rises (25.7%) at scores 7–9 and has a high probability (91.8%) of being a pulmonary metastasis at scores 10–15.

**Table 4:** Pulmonary Metastasis Prediction Model scale for patients with IPNs after radical esophagectomy

Predictive marker	Model points
**Pathologic N category**	
0	0
1	1
2	2
3	3
**Number of IPNs**	
< 3	0
≥ 3	3
**Shape of the largest IPN**	
Round or round-like	2
Others	0
**Time of detection of IPNs (years)**	
< 1	0
1–2	1
> 2	2
**Size of the largest IPN (mm)**	
< 5	0
5–10	3
10–30	4
> 30	6

**Table 5:** Probability of pulmonary metastasis based on the Pulmonary Metastasis Prediction Model scale in 221 patients with IPNs

Total score	0–6	7–9	10–15
Pulmonary metastases rate	0.8%	25.7%	91.8%

## Discussion

The presence of distant organ metastases is a poor prognostic factor for most malignancies; however, the natural course of this process varies from cancer to cancer [17]. Depending on the origin, cell subtype, and tissue affinity of the tumor, metastases may appear rapidly in multiple organs or in specific organs after a long latency period [10, 18–20]. The fact that the lungs are rich in blood vessels and lymphatic vessels makes them a common site for hematogenous metastasis [4, 5, 10]. The rate of pulmonary metastases is high; so, close monitoring of pulmonary metastases and their diagnosis and treatment at an early stage of the disease, especially the early treatment of resectable pulmonary metastases, is crucial and can significantly improve their survival rates [21, 22].

Pulmonary metastases may only present as single or multiple pulmonary nodules that are difficult to distinguish from non-metastatic lesions in the early stages, and the high incidence of non-metastatic lesions in the lung further increases the difficulty of the early diagnosis of pulmonary metastases [8]. The current diagnostic standard for the postoperative pulmonary metastasis of esophageal cancer is still biopsy with histopathology; however, it is not suitable for the detection and diagnosis of early lesions due to its invasive nature and low accuracy in the early stages [23, 24]. Only a few studies have suggested risk factors for pulmonary metastasis after esophageal cancer surgery [25, 26]; however, guidelines such as those of the NCCN and AJCC and large-scale studies with high levels of evidence do not suggest how to determine and manage pulmonary nodules after esophageal cancer surgery [12, 13]. To enhance our understanding of the clinical impact of the pulmonary metastasis of esophageal cancer, we analyzed the clinical characteristics and patterns of 221 patients with lung metastases from esophageal cancer and expected to build a predictive model based on them that could help guide clinical decision-making. To the best of our knowledge, this is the largest study on esophageal cancer with lung metastasis.

The relevant literature indicates that in extra-pulmonary malignancies, depending on the primary tumor and the type of pathology, approximately 30–70% of patients present with unidentified pulmonary nodules, and of these unidentified pulmonary nodules, approximately 30% are diagnosed as metastatic lesions [8, 27]. In the present study, the incidence of IPNs was 27.1%, and 29.9% of patients with IPNs were diagnosed with pulmonary metastasis during follow-up. The typical radiological findings of pulmonary metastases include one or more round nodules of variable sizes located in the periphery [28, 29]. Also, in our study, we found that almost all pulmonary metastatic nodules from esophageal squamous carcinoma fit the above description and appeared as round or round-like, solid lesions without calcifications on CT.

Pulmonary metastasis, as the most common site of metastasis after surgery for esophageal squamous carcinoma, significantly affects the prognosis and quality of life of patients. However, pulmonary metastases are different from other organ metastases, and new IPNs that appear after surgery are not necessarily metastatic lesions; instead, non-metastatic lesions are the majority, accounting for approximately 70%. Moreover, biopsy with histopathology is still the gold standard for pulmonary metastases, and samples for histopathology were obtained mainly via percutaneous lung aspiration biopsy or thoracoscopic resection biopsy, and thoracoscopic biopsy is less commonly used clinically because of its high invasiveness. However, in our study, there were only 102 (46.2%) postoperative IPNs located in the outer 1/3 of the lung field. Puncture of pulmonary nodules in the middle and inner 1/3 of the lung field is more difficult and associated with significantly more puncture-related complications [23]. Also, diagnostic thoracoscopic partial lung resection is not usually recommended for pulmonary nodules in the middle and inner 1/3 of the lung field due to the large extent of resection. At this time, the diagnosis of new unidentified lesions in the lung is mainly based on the clinician’s experience; so, it is more common to miss and misdiagnose lung metastases.

There are only a few studies in the literature on risk factors for postoperative pulmonary metastasis in squamous esophageal cancer. Ai et al. showed that squamous cell carcinoma was a risk factor for the postoperative pulmonary metastasis of esophageal cancer while general characteristics such as age and gender and clinical characteristics such as the pathological *T* stage and pathological *N* stage were not significantly associated with non-metastasis [26]. In our study, the results of a multivariate analysis showed that the pathologic N category, number of IPNs, shape of the largest IPN, time of detection of IPNs, and size of the largest IPN were independent risk factors for the pulmonary metastasis of esophageal cancer. We constructed a non-invasive diagnostic criteria model (PMPM scale) for postoperative IPNs in esophageal cancer based on multivariate analysis results. After validated, the PMPM scale showed good discrimination with an AUC of 0.939. When patients with IPN nodules are scored 1–6 by the PMPM scale, there is a low likelihood of pulmonary metastases and observation is recommended. When the score is 7–9, there is a 25.7% chance of pulmonary metastases and further examination or increased follow-up is recommended. When scored 10–15, there is a high probability of metastatic lesions and positive clinical intervention is recommended for diagnosis and further treatment.

To the best of our knowledge, this is the first study to propose a model for the diagnosis of IPNs after esophageal cancer surgery. We hope that this model will be further refined in the future to aid clinical decision-making. The primary limitations of this study include its retrospective, single-center design and the absence of external validation. The findings will need to be confirmed in future multi-center, larger-scale, and prospective studies. In summary, we established and validated a clinical model that accurately identifies pulmonary metastasis from IPNs after esophagectomy.

## Data Availability

The data presented in this study are available on request from the corresponding author.

## Abbreviations

IPNs: Indeterminate pulmonary nodules
ESCC: Esophageal squamous cell carcinoma
PMPM: Pulmonary Metastasis Prediction Model
